# Relationship Between Attention Deficit Hyperactivity Disorder and Alcohol Dependence: A Genetic View

**DOI:** 10.5812/ijhrba.9629

**Published:** 2013-03-12

**Authors:** Ke-Sheng Wang

**Affiliations:** 1 Department of Biostatistics and Epidemiology, College of Public Health, East Tennessee State University, Johnson City, USA

**Keywords:** Attention Deficit-Hyperactivity Disorder, Alcohol Dependence, Genetics

*Dear Editor,*

Attention deficit hyperactivity disorder (ADHD), is a common, highly heritable childhood-onset psychiatric disorder affecting 2% – 6% of children worldwide. The recent paper examines the effects of child and adult ADHD symptoms in adults among male students on adult addiction acknowledgment and alcoholism potential (1). This article is an original study which provides that adult ADHD and child ADHD predict addiction acknowledgment while child ADHD and impulsivity predict alcohol potential. These findings add new evidence about prediction of adolescent and adult drug abuse and use by early ADHD. However, the biological mechanism of the relationship between ADHD and substance problems is still unclear. One of the important mechanisms is genetic correlation due to shared genetic loci. First, it has been shown that both ADHD and alcohol dependence have strong genetics components. Twin and adoption studies have indicated that ADHD has high heritability in the range of 75% – 91% (2), while family, twin, and adoption studies have revealed that genetic and environmental factors and their interactions contribute to the development of alcohol dependence, with a heritability of more than 50% (3). Second, ADHD has been found to be highly comorbid with nicotine dependence and alcohol dependence. Segregation analysis has provided multigenerational evidence of cosegregation among ADHD, nicotine dependence, and alcohol dependence, while linkage analysis has suggested several regions on human chromosomes with pleiotropic effects on ADHD and alcohol dependence (4). For example, significant linkages at 4q13.1 - 13.2 and 5q31 - 33 for ADHD has been implicated in alcohol dependence and nicotine dependence (4). Third, genetic association studies have suggested several genes which are responsible for both ADHD and alcohol dependence. For example, it has been reported associations between the dopamine transporter (DAT) gene polymorphisms and human disorders including ADHD and alcohol dependence (5). The variants of the dopamine D2 receptor gene (DRD2) has been found to be associated with alcoholism, drug dependency, obesity, smoking, pathological gambling, ADHD, Tourette syndrome, as well as other related compulsive behaviors (6). The tachykinin receptor 1 (TACR1) gene has been reported to be associated with bipolar disorder and alcoholism as well as ADHD (2). However, parts of the associations are not strong and the results are not always consistent due to biologic mechanisms (genetic heterogeneity, gene-gene interactions and gene-environment interactions etc.) and spurious mechanisms (inadequacies of genomic markers, type 1 error, limited sample sizes and power, cohort and age effects, and bias etc.). In addition, some other genes, such as the nicotinic receptor CHRNA5-A3-B4 subunit genes, do not contribute to the common genetic predisposition of ADHD and alcohol dependence (2, 7).

In short, the current results not only support previous studies about the relationship between ADHD and alcohol and drug dependence but also provide further evidence on prediction of future substance dependence in adulthood through early ADHD (1). However, the sample size for the current study is relative small; therefore the results need further investigation using large sample and confirmation in other populations. In addition, further genetic and epigenetic study to identify the disease-causing variants including gene-environment interactions in ADHD and alcohol dependence would have promise for understanding the pathogenesis of these comorbity psychiatric disorders, predicting the risks and developing potential treatments for ADHD and alcohol dependence.
